# Percutaneous CT-Guided Cryoablation for Pain Palliation and Local Treatment Effect in Unresectable Pancreatic Ductal Adenocarcinoma: A Pilot Single-Center Case Series

**DOI:** 10.3390/cancers18111724

**Published:** 2026-05-25

**Authors:** Claudio Pusceddu, Claudio Carrubba, Pierluigi Maria Rinaldi, Claudio Cau, Felice D’Antuono, Francesco Giurazza, Raffaella Niola, Salvatore Marsico

**Affiliations:** 1 Department of Radiology, Mater Olbia Hospital, SS 125 Orientale Sarda, 07026 Olbia, Italy; clapusceddu@gmail.com (C.P.); pierluigi.rinaldi@materolbia.com (P.M.R.); claudio.cau@materolbia.com (C.C.); 2 Vascular and Interventional Radiology Department, Cardarelli Hospital, Via Antonio Cardarelli 9, 80131 Naples, Italy; felice.dantuono@aocardarelli.it (F.D.); francesco.giurazza@aocardarelli.it (F.G.); raffaella.niola@aocardarelli.it (R.N.); 3 Department of Radiology, Hospital del Mar, Passeig Marítim 25, 08003 Barcelona, Spain; salvatore.marsico@hotmail.it

**Keywords:** pancreatic ductal adenocarcinoma, cryoablation, pain palliation, interventional radiology, opioid reduction, pancreatic cancer

## Abstract

**Background/Objectives:** Pain is one of the most disabling symptoms in patients with unresectable pancreatic ductal adenocarcinoma (PDAC), having a major impact on quality of life, functional status, and tolerance to oncologic treatment. Percutaneous computed tomography (CT)-guided cryoablation may provide tumor-directed pain palliation in selected patients. This study aimed to evaluate the safety and palliative clinical benefit of percutaneous CT-guided cryoablation in patients with painful unresectable PDAC. **Methods:** This retrospective single-center pilot case series included 11 consecutive patients with painful unresectable PDAC treated with percutaneous CT-guided cryoablation between January 2022 and May 2024. Primary endpoints were change in visual analogue scale (VAS) score and reduction in analgesic requirement. Secondary endpoints included technical success, adverse events, supportive clinical outcomes, imaging evolution, progression status, and survival. **Results:** Technical success was achieved in all procedures (11/11, 100%). No major procedure-related complications occurred; minor adverse events were observed in 3/11 patients (27.3%). Mean VAS score decreased from 6.72 ± 1.56 at baseline to 3.45 ± 1.44 at 1 month, 2.54 ± 1.29 at 3 months, 2.27 ± 1.43 at 6 months, and 1.60 ± 1.07 at 12 months. At 1 month, all patients showed a reduction of at least 3 VAS points. A reduction in analgesic requirement was documented in all patients during early follow-up, with complete opioid discontinuation in 5/11 patients (45.5%). At 1-month CT, residual enhancement was present in 9/11 patients (81.8%), although with an estimated 50–80% reduction in enhancing tumor burden. Observed survival proportions at 6 and 12 months were 90.9% and 72.7%, respectively. **Conclusions:** Percutaneous CT-guided cryoablation appears to be a feasible palliative option for selected patients with painful unresectable PDAC, with meaningful pain relief, opioid sparing, acceptable short-term safety, and exploratory imaging evidence of local cytoreductive effect. Further prospective studies are warranted.

## Simple Summary

Pain is a major problem in patients with unresectable pancreatic cancer, and often requires opioid treatment, which may reduce quality of life. This pilot single-center case series evaluated computed tomography-guided cryoablation as a palliative, tumor-directed treatment in 11 patients with painful unresectable pancreatic ductal adenocarcinoma. The procedure was technically successful in all patients, and no major complications were observed. Pain intensity decreased after treatment, and all patients showed a reduction in opioid requirement during early follow-up. These preliminary findings suggest that computed tomography-guided cryoablation may be a feasible palliative option in selected patients with painful unresectable pancreatic cancer and may support further prospective research in this field.

## 1. Introduction

Pancreatic ductal adenocarcinoma (PDAC) remains one of the most aggressive malignancies worldwide and is associated with a poor prognosis [1]. At diagnosis, most patients present with either locally advanced unresectable or metastatic disease, and only a minority are candidates for potentially curative surgical resection [1,2]. In patients with unresectable disease, survival remains limited despite recent advances in systemic chemotherapy and radiotherapy [2,3,4].

Pain is one of the most frequent and disabling symptoms in PDAC and has a major impact on quality of life, performance status, and tolerance to oncologic treatment [5,6]. The mechanisms of pancreatic cancer-related pain are complex and include local tumor invasion, perineural infiltration, ductal obstruction, and inflammatory changes [5,6]. Therefore, effective pain control represents a crucial component of the multidisciplinary management of patients with advanced pancreatic cancer.

Current guidelines support a multimodal approach for pain palliation, including opioid-based analgesia, celiac plexus neurolysis, radiation therapy, and other supportive or interventional strategies [7,8]. Among these, celiac plexus neurolysis is the most widely adopted interventional option for visceral pain relief [2,7]. However, its efficacy may be limited in both magnitude and duration, and clinical benefit is not always satisfactory, particularly in advanced disease with plexus infiltration [9,10,11].

In this setting, local ablative therapies directed at the tumor itself have gained growing interest as a complementary strategy for symptom control and local disease management [12,13,14,15,16]. Several techniques, including radiofrequency ablation, irreversible electroporation, microwave ablation, and cryoablation, have been investigated in locally advanced pancreatic cancer, although the available evidence remains limited and heterogeneous [13,14,15,16]. Although a clear survival advantage has not been demonstrated, these procedures may offer palliative benefit through local cytoreduction, reduction in tumor-related inflammation, and possible decompression of adjacent neural structures. Tumor-directed local therapies may therefore be particularly attractive because they may combine symptom palliation with local treatment effects in patients who are not candidates for curative treatment [4,15,16].

Cryoablation induces tumor destruction through repeated freeze–thaw cycles, leading to extracellular and intracellular ice formation, vascular injury, and coagulative necrosis [17]. For pancreatic applications, this technique may offer some practical advantages, including clear visualization of the ice ball on computed tomography (CT), real-time monitoring during the procedure, and a potentially favorable safety profile in selected patients [17]. Early clinical experiences with pancreatic cryoablation, first in intraoperative settings and later with percutaneous approaches, suggested that this technique may be feasible and may contribute to pain relief and local treatment effect in unresectable disease [18,19,20,21,22]. Kovach et al. reported early surgical experience in unresectable pancreatic cancer [18], while Niu et al. described percutaneous cryoablation with encouraging results in terms of pain reduction and decreased analgesic consumption [19,22]. Other authors have explored cryoablation in combination with brachytherapy or bypass surgery, further supporting the potential palliative role of this approach in selected patients [20,21].

Despite these preliminary findings, the current literature on pancreatic cryoablation is still limited to small retrospective series, with heterogeneous populations, mixed techniques, and non-uniform clinical endpoints [17,18,19,20,21,22].

Recent reviews on thermal ablation in pancreatic cancer have further highlighted the growing interest in image-guided ablative therapies for patients who are not candidates for surgical resection, while emphasizing the persistent heterogeneity of available evidence and the need for standardized protocols and prospective studies [12,23]. In parallel, technological developments, including prototype cryoablation needle systems designed for potential compatibility with endoscopic ultrasound guidance, may expand future applications of cryoablation in pancreatic tumors, although these data remain preliminary and largely preclinical [24]. Moreover, cryoablation has been increasingly investigated for its potential immunomodulatory effects, including tumor antigen release and stimulation of antitumor immune responses, opening possible perspectives for combination strategies with systemic or immunotherapeutic treatments [25].

In particular, data specifically focused on percutaneous CT-guided cryoablation as a palliative tumor-directed treatment in patients with painful unresectable PDAC remain scarce, especially with regard to the combined assessment of pain relief, analgesic requirement, supportive clinical outcomes, and local imaging evolution over time.

The aim of this study was to retrospectively evaluate the safety and palliative clinical benefit of percutaneous CT-guided cryoablation in patients with painful unresectable PDAC, with a primary focus on pain response and reduction in analgesic requirement, while also exploring its potential role as a tumor-directed palliative treatment through the assessment of supportive clinical outcomes, local imaging evolution, and survival.

## 2. Materials and Methods

### 2.1. Study Design and Patient Selection

This retrospective single-center pilot case series included consecutive patients with painful unresectable pancreatic ductal adenocarcinoma (PDAC) who underwent percutaneous CT-guided cryoablation with palliative intent at Mater Olbia Hospital, Olbia, Italy, between January 2022 and May 2024.

This study was conducted in accordance with the Declaration of Helsinki and was approved by the Institutional Ethics Committee of our institution. Written informed consent was obtained from all participants before enrollment. The study population was identified from our institutional database of interventional oncology procedures.

Eligibility required histologically confirmed pancreatic ductal adenocarcinoma. Only primary pancreatic adenocarcinomas were included. Patients with non-ductal pancreatic tumors or secondary pancreatic tumors were excluded. A total of 11 patients were included (1 man and 10 women). All patients had unresectable disease at the time of treatment. Unresectability was determined at multidisciplinary tumor board review based on radiological extent of disease and overall clinical status. Only one patient had previously undergone surgery and was treated for local recurrence, whereas the remaining patients had unresectable primary disease.

At the time of cryoablation, disease extent was classified as either locally advanced unresectable or metastatic unresectable. Tumor location, maximum axial tumor diameter, vascular involvement, and metastatic status were recorded from baseline contrast-enhanced CT imaging. Patients with direct invasion of the duodenum or other adjacent hollow viscera were excluded from treatment in order to reduce the risk of major procedure-related complications.

In addition to histologic confirmation and clinical unresectability, patient selection was guided by local institutional criteria, including ECOG performance status ≤ 3, platelet count > 50 × 10^9^/L, INR < 1.5, and maximum tumor diameter < 6 cm.

Previous and concomitant oncologic treatments were recorded, including prior surgery, prior chemotherapy, ongoing chemotherapy at the time of cryoablation, and prior radiotherapy.

### 2.2. Pre-Procedural Pain Assessment and Supportive Clinical Evaluation

Before cryoablation, all patients underwent clinical assessment focused on pain burden and pain-related functional impairment. Pain intensity was assessed using a 10-point visual analogue scale (VAS), where 0 indicated no pain and 10 indicated the worst pain imaginable. Baseline VAS score was recorded before cryoablation, and follow-up VAS scores were collected during scheduled clinical assessments at 1, 3, 6, and 12 months whenever available. Because of the retrospective design and patient attrition during follow-up, VAS data were not available for all patients at every time point; therefore, analyses were performed only in evaluable patients with available paired data. Pain was considered tumor-related according to multidisciplinary clinical evaluation, taking into account disease location, imaging findings, and symptom pattern. Only patients with moderate-to-severe tumor-related pain were included. In this study, baseline VAS scores ranged from 5 to 10.

Because of the retrospective design, analgesic requirement was assessed qualitatively from clinical records rather than quantitatively using standardized morphine-equivalent daily dose. At baseline, patients were receiving opioid-based analgesic therapy according to pain severity and clinical need. During follow-up, analgesic requirement was categorized according to documented complete opioid discontinuation, opioid reduction without discontinuation, unchanged analgesic treatment, or escalation of analgesic therapy.

Supportive clinical evaluation included ECOG performance status, body weight, and sleep quality.

Sleep quality was not assessed using a validated instrument; because of the retrospective design, it was derived from chart review and categorized descriptively as improved, unchanged, or worsened compared with baseline.

### 2.3. Cryoablation Procedure

All cryoablation procedures were performed percutaneously under CT guidance and conscious sedation by experienced interventional radiologists. Procedures were performed using a multidetector CT system. Before probe placement, an initial non-contrast CT scan was obtained to confirm tumor location, patient positioning, and the safest access route. When required, contrast-enhanced CT images were reviewed or acquired to better define the relationship between the target lesion and adjacent vascular, biliary, gastric, and duodenal structures. Local anesthesia was administered at the skin entry site, and conscious sedation was performed according to institutional practice. Sedation depth and analgesic support were tailored to patient tolerance and procedural duration, with continuous clinical monitoring throughout the procedure.

Patients were treated in the supine position. Access route was selected according to tumor location and the safest available needle trajectory. A transgastric approach was adopted in selected cases when lesion location and surrounding anatomy made a conventional transabdominal route less favorable. Probe number was selected according to maximum tumor diameter: 1 probe for lesions <3 cm, 2 probes for lesions measuring 3–4 cm, and 3 probes for lesions >4 cm. Cryoablation was performed using IceRod™ and IceForce™ cryoablation needles (Boston Scientific, Marlborough, MA, USA).

During probe advancement, intermittent CT scans were obtained to verify needle trajectory, probe orientation, and final cryoprobe position within the target lesion. Intraprocedural CT acquisitions were also performed during the freezing cycles to monitor ice-ball formation and evaluate its relationship with adjacent critical structures, including the duodenum, stomach, and major vessels.

The ablation zone was monitored dynamically during each freezing cycle, and the procedure was continued only when the ice-ball configuration was considered compatible with adequate lesion coverage and preservation of adjacent critical structures. At the end of the procedure, a final CT scan was obtained to assess the immediate technical result and exclude early procedure-related complications, including bleeding, pancreatitis-related fluid collections, pneumoperitoneum, or injury to adjacent hollow viscera.

The procedural objective was palliative local cytoreduction for pain relief rather than wide-margin oncologic ablation. No celiac plexus or splanchnic nerve cryoablation/cryo-neurolysis was performed in this series. The procedure was intentionally conceived as a palliative intratumoral treatment strategy rather than a radical margin-oriented ablation, in order to balance symptom control, local cytoreduction, and procedural safety. For this reason, cryoablation was intentionally confined to the target lesion without attempting ablation beyond tumor margins, in order to reduce the risk of pancreatitis and injury to adjacent critical structures.

A standard double freeze–thaw cycle was used. Each cycle consisted of 10 min of freezing followed by 10 min of thawing, and the sequence was then repeated. No hydrodissection or other adjunctive protective displacement techniques were required in any case. Technical success was defined as correct probe placement according to the treatment plan and completion of the intended cryoablation cycle.

### 2.4. Post-Procedural Care

After the procedure, patients were hospitalized for post-procedural observation according to clinical status, with a length of stay ranging from 1 to 5 days. During this period, vital signs and overall clinical condition were monitored, and patients were evaluated for early complications. Post-procedural care consisted of standard supportive management. Procedure-related complications were assessed clinically and, when indicated, radiologically.

Adverse events were recorded both per patient and per event, with particular attention to pancreatitis, bleeding, duodenal injury, infection, and worsening abdominal pain.

### 2.5. Follow-Up Protocol

Patients underwent scheduled clinical and imaging follow-up after cryoablation.

Clinical follow-up was scheduled at 1, 3, 6, and 12 months after cryoablation, whereas contrast-enhanced CT was routinely performed at 1, 6, and 12 months, with specific attention to residual enhancement and local progression over time.

At each follow-up time point, the following variables were assessed whenever available:

- VAS score;
- Analgesic requirement;
- ECOG performance status;
- Body weight;
- Sleep quality;
- Imaging findings;
- Disease progression status;
- Survival status.

For each follow-up time point, analyses were performed on evaluable patients with available clinical or imaging data. Missing data were not imputed, and patients were included in each analysis up to the last available follow-up assessment. Patients who died before completion of the 12-month follow-up were censored for subsequent time points and included in the analysis up to the time of death.

### 2.6. Study Endpoints

The primary endpoints were

1. Change in VAS score from baseline.
2. Reduction in analgesic requirement after cryoablation.

The secondary endpoints were

1. Change in ECOG performance status.
2. Body weight improvement, including weight gain >5% from baseline.
3. Change in sleep quality.
4. Imaging-based local assessments at 1, 6, and 12 months.
5. Progression status and survival during follow-up.

A clinically meaningful pain response was defined as a reduction of at least 2 points in VAS score compared with baseline. The study was designed primarily as a palliative clinical assessment; imaging findings were therefore considered supportive secondary outcomes rather than the main objective of the study.

Imaging outcomes were assessed descriptively on follow-up contrast-enhanced CT, focusing on changes in residual enhancement, necrotic appearance, and local morphological evolution of the treated lesion. Standardized response criteria, including RECIST, mRECIST, or Choi criteria, were not applied.

### 2.7. Statistical Analysis

Continuous variables were summarized as mean ± standard deviation or median and interquartile range, as appropriate, while categorical variables were reported as counts and percentages. Given the small sample size and pilot nature of the study, all analyses were considered exploratory and hypothesis-generating. Changes in VAS scores over time were assessed using the Wilcoxon signed-rank test for paired samples, comparing each follow-up time point with baseline in evaluable patients. No imputation of missing data was performed. Given the exploratory nature of this pilot series and the small sample size, *p*-values were interpreted descriptively and were not intended to support definitive hypothesis testing. Emphasis was placed on the magnitude and clinical consistency of pain reduction rather than on statistical significance alone.

## 3. Results

### 3.1. Patient Characteristics

Between January 2022 and May 2024, 11 consecutive patients with painful unresectable pancreatic ductal adenocarcinoma underwent percutaneous CT-guided cryoablation with palliative intent. The study included 10 women and 1 man; all patients were treated during the study period.

At baseline, 5/11 patients (45.5%) had an ECOG performance status of 2, while 6/11 (54.5%) had an ECOG performance status of 3.

Baseline weight loss was documented in 9/11 patients (81.8%), while 6/11 (54.5%) reported sleep disturbance related to tumor-associated pain. Vascular infiltration was observed in 8/11 patients (72.7%), and metastatic disease was present in 9/11 (81.8%). Mean maximum axial tumor diameter was 39.36 ± 9.81 mm. Tumors were located in the pancreatic head in 8/11 patients (72.7%) and in the pancreatic body/tail region in 3/11 patients (27.3%).

Baseline pain burden was clinically relevant in all patients, with a mean VAS score of 6.72 ± 1.56. All patients (100%) were receiving opioid-based analgesic therapy before cryoablation.

With regard to previous oncologic treatments, 1/11 patients (9.1%) had undergone prior surgery for local recurrence, 8/11 (72.7%) had received chemotherapy, and 2/11 (18.2%) had previously undergone radiotherapy. Details of previous and concomitant oncologic treatments are summarized in Table 1.

### 3.2. Procedural Outcomes

All cryoablation procedures were completed as planned under CT guidance and analgosedation. Technical success was achieved in all cases (11/11, 100%). A transgastric approach was required in 2/11 procedures (18.2%), whereas the remaining cases were treated through a conventional percutaneous transabdominal route.

No major procedure-related complications were observed. Minor post-procedural adverse events occurred in 3/11 patients (27.3%), accounting for a total of 5 events. Two patients experienced transient abdominal pain, which resolved spontaneously within 48–72 h, two patients reported self-limited nausea, and one patient developed a pleural effusion that was managed conservatively. No clinically overt post-procedural pancreatitis, bleeding, duodenal injury, infection, or procedure-related death was recorded.

### 3.3. Pain Response

A clinically meaningful reduction in pain intensity was documented across the cohort after cryoablation.

**Figure 1:**
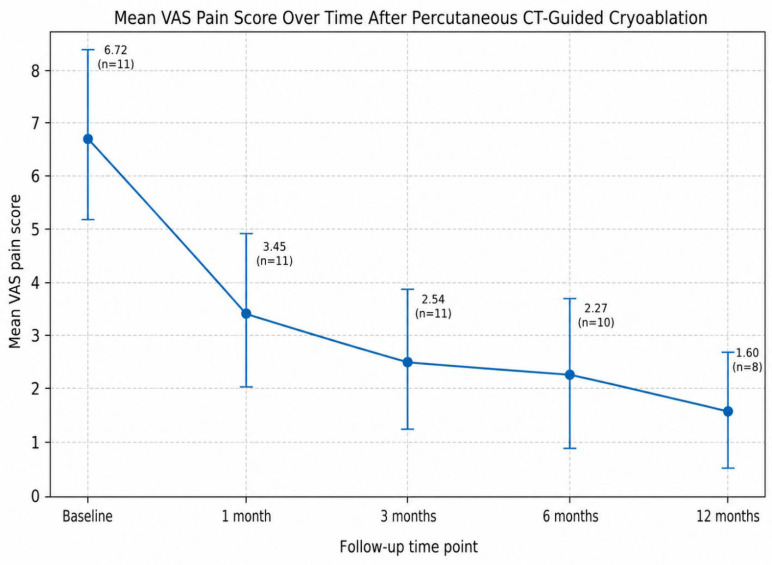
Mean VAS score over time after percutaneous CT-guided cryoablation. Longitudinal trend of mean visual analogue scale (VAS) scores at baseline and during follow-up after percutaneous CT-guided cryoablation. Error bars indicate standard deviation. Numbers above each time point indicate mean VAS values and the number of evaluable patients. A rapid reduction in pain intensity was observed at 1 month and was maintained throughout follow-up.

At 1 month, all 11/11 evaluable patients demonstrated a reduction in VAS score of at least 3 points compared with baseline. Mean VAS decreased from 6.72 ± 1.56 at baseline to 3.45 ± 1.44 at 1 month (*p* = 0.002, Wilcoxon signed-rank test). At 3 months, mean VAS further declined to 2.54 ± 1.29 among 11/11 evaluable patients (*p* = 0.001 vs. baseline). At 6 months, mean VAS remained low at 2.27 ± 1.43 among 10/11 evaluable patients (*p* = 0.001 vs. baseline). At 12 months, among 8/11 evaluable patients, mean VAS was 1.60 ± 1.07 (*p* = 0.031 vs. baseline; Figure 1).

### 3.4. Analgesic Requirement

A reduction in analgesic requirement was documented during early follow-up based on qualitative review of clinical records. At baseline, all 11/11 patients were receiving opioid-based analgesic therapy. By 1 month, 5/11 patients (45.5%) had achieved complete discontinuation of opioid therapy, whereas the remaining 6/11 patients (54.5%) had documented opioid reduction without complete discontinuation. No patient required escalation of pain medication during early follow-up. Standardized morphine-equivalent daily doses were not systematically available.

### 3.5. Supportive Clinical Outcomes

Among the 9 patients who had baseline weight loss, 6 (66.7%) experienced subsequent weight recovery, with a gain exceeding 5% of baseline body weight during follow-up.

Sleep disturbance was present in 6/11 patients (54.5%) before treatment. Of these, 3/6 patients (50.0%) reported clinically appreciable improvement in sleep quality after cryoablation, while the remaining patients had either stable symptoms or incomplete recovery.

At 1 month, ECOG performance status improved by at least one level in 4/11 patients (36.4%), remained stable in 6/11 (54.5%), and worsened in 1/11 (9.1%). The distribution of ECOG scores at 1 month was as follows: ECOG 1 in 1/11 patients (9.1%), ECOG 2 in 7/11 (63.6%), and ECOG 3 in 3/11 (27.3%).

Among patients who remained alive and clinically evaluable, ECOG improvement was generally maintained at 3 and 6 months, whereas late functional deterioration was mainly observed in patients with systemic disease progression.

These findings should be interpreted cautiously, as supportive clinical outcomes were assessed retrospectively and without standardized validated instruments, but they may be suggestive of broader clinical benefit after cryoablation.

### 3.6. Imaging Findings

At the 1-month contrast-enhanced CT assessment, residual enhancement was observed in 9/11 patients (81.8%), although with an estimated 50–80% reduction in enhancing tumor burden compared with baseline. In 2/11 patients (18.2%), no residual enhancement was appreciable. These findings were interpreted descriptively as an exploratory local cytoreductive effect at the treated site, reflected by reduction in the contrast-enhancing component and in line with the intentionally non-radical and palliative nature of the procedure.

At the 6-month imaging follow-up, 10/11 patients were evaluable, as one patient had died before this time point. Among evaluable patients, CT was used to assess persistence of local cytoreduction and disease evolution; systemic progression was documented in 3 patients by 6 months.

At the 12-month imaging follow-up, 8/11 patients were evaluable, as three patients had died before the 1-year assessment. In the remaining evaluable patients, imaging was primarily used to monitor maintenance of local tumor reduction and overall disease course. No patient underwent repeat local treatment during follow-up.

**Figure 2:**
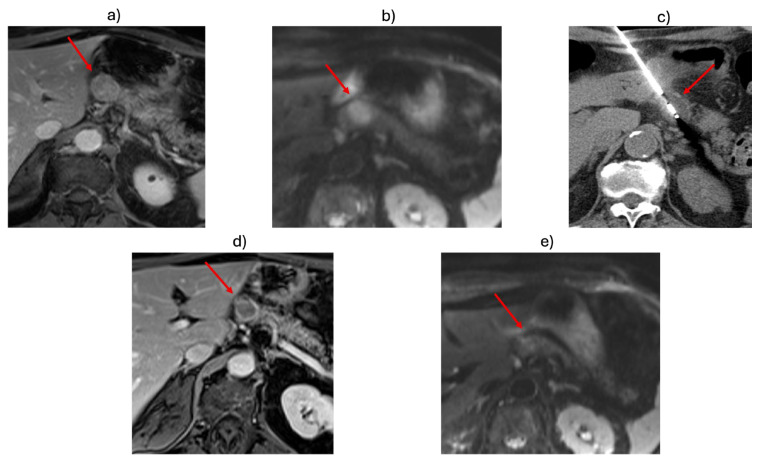
Representative case of transgastric CT-guided cryoablation for pancreatic head adenocarcinoma. A representative case of painful unresectable pancreatic head adenocarcinoma treated with percutaneous CT-guided cryoablation through a transgastric approach. (**a**) Pre-procedural contrast-enhanced CT showing the pancreatic head lesion. (**b**) Pre-procedural MRI with diffusion-weighted imaging (DWI) confirming the lesion. (**c**) Intraprocedural CT image obtained during transgastric cryoablation. (**d**) Contrast-enhanced CT at 12-month follow-up. (**e**) Follow-up DWI-MRI at 12 months showing no appreciable residual diffusion restriction at the treated site. Arrows indicate the target lesion, cryoprobe position, and corresponding treated area during follow-up. Follow-up imaging demonstrates sustained reduction in the treated lesion, consistent with a local cytoreductive effect after cryoablation.

**Figure 3:**
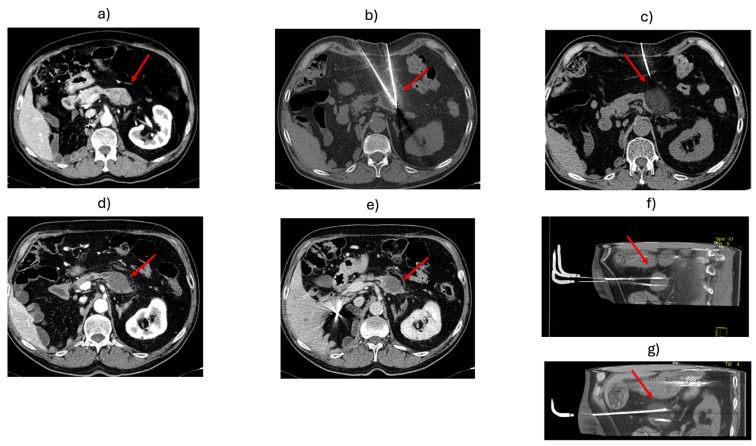
Representative case of CT-guided percutaneous cryoablation for pancreatic body adenocarcinoma. Representative case of painful unresectable pancreatic body adenocarcinoma treated with percutaneous CT-guided cryoablation. (**a**) Pre-procedural contrast-enhanced CT showing the pancreatic body lesion. (**b**,**c**) Axial intraprocedural CT images showing positioning of the cryoprobes within the target lesion. (**d**) Contrast-enhanced CT at 1-month follow-up. (**e**) Contrast-enhanced CT at 4-month follow-up. (**f**,**g**) Sagittal intraprocedural CT images showing positioning of the two cryoprobes along the longitudinal axis of the lesion. Arrows indicate the pancreatic lesion before treatment, the procedural target/cryoprobe position during cryoablation, and the corresponding treated area during follow-up. Follow-up imaging demonstrates persistent reduction in the contrast-enhancing component at the treated site after cryoablation.

Overall, imaging findings provided supportive and exploratory evidence of a local cytoreductive effect in the treated area, mainly reflected by reduction in the contrast-enhancing tumor component, in keeping with the tumor-directed palliative intent of the procedure. (Figure 2 and Figure 3).

### 3.7. Disease Progression and Observed Survival

Systemic disease progression was documented in 3/11 patients (27.3%) at 6 months. No patient underwent repeat cryoablation or any additional local retreatment of the pancreatic lesion during the study period. By 6 months, 1 patient had died; by 12 months, 3 deaths had occurred in total. Observed survival proportions at 6 and 12 months were 90.9% and 72.7%, respectively. Median overall survival was not reached during the available follow-up period. (Table 2).

## 4. Discussion

This pilot case series suggests that percutaneous CT-guided cryoablation may represent a feasible, safe, and clinically useful palliative option for patients with painful unresectable PDAC. In our series, all procedures were technically successful, no major complications were observed, and all patients experienced a clinically meaningful reduction in pain intensity after treatment. In addition, cryoablation was associated with a substantial reduction in opioid requirement and with supportive clinical benefit in a subset of patients, including improvement in performance status, sleep quality, and body weight.

Pain control is one of the main goals in the management of advanced pancreatic cancer, since pain has a major impact on quality of life, nutritional status, and tolerance to oncologic treatment [6,7,8]. In our study, mean VAS score progressively decreased from 6.72 at baseline to 3.45 at 1 month, 2.54 at 3 months, 2.27 at 6 months, and 1.60 at 12 months in evaluable patients. All patients achieved a reduction of at least 3 VAS points at 1 month, supporting the rapid palliative effect of the procedure.

These findings are consistent with the palliative intent of the procedure and are in line with previous reports describing pain reduction after pancreatic cryoablation. However, because of the retrospective design, small sample size, and absence of a control group, no direct comparison can be made with established palliative strategies such as celiac plexus neurolysis [2,7,9,10,11], celiac plexus radiosurgery, systemic therapy, or optimized medical pain management [26,27,28,29]. Moreover, the observed pain reduction may have been influenced by several confounding factors, including the natural variability of cancer-related pain, concomitant oncologic and supportive treatments, patient selection, and possible placebo- or procedure-related effects. Therefore, the present findings should be interpreted as preliminary associations rather than definitive evidence of treatment efficacy.

The palliative effect observed in our series is in line with previous reports on pancreatic cryoablation and with broader evidence supporting cryoablation for cancer pain palliation [30]. Early experience by Kovach et al. showed that cryoablation could be performed safely in unresectable pancreatic cancer, with good pain control at discharge [18]. Niu et al. later reported encouraging results with percutaneous cryoablation, including reduction in pain scores and analgesic consumption [19]. In a subsequent series, the same group suggested that cryoablation combined with celiac plexus block could further improve visceral pain control [22]. Other authors also explored cryoablation in combination with brachytherapy or palliative bypass surgery, supporting a possible palliative role for cryo-based tumor-directed treatment in selected patients with advanced disease [20,21]. Our findings are consistent with this literature, but differ in one important aspect: in the present study, percutaneous CT-guided cryoablation was primarily used as a palliative tumor-directed intervention specifically focused on pain control in painful unresectable PDAC.

Patient selection should also be interpreted in the context of the heterogeneous clinical background of this cohort. Most patients had metastatic unresectable disease, and previous or concomitant oncologic treatments varied across the study population. These factors may have influenced baseline symptom burden, pain trajectory, functional status, and clinical outcomes after cryoablation. Because of the small sample size, formal subgroup analyses according to disease extent or treatment history were not feasible. Therefore, the present results should be considered descriptive and hypothesis-generating rather than definitive evidence applicable to all patients with unresectable PDAC.

In our multidisciplinary practice, cryoablation was selected as a tumor-directed palliative option in carefully selected patients with persistent tumor-related pain, in whom local tumor burden was considered a relevant contributor to symptoms and in whom the anatomical relationship between the tumor and adjacent critical structures allowed for a safe percutaneous approach. The procedure was not intended to replace established palliative strategies, but rather to complement systemic, analgesic, and supportive treatments in selected cases.

While celiac plexus neurolysis targets the neural pathway of pancreatic cancer-related pain, tumor-directed cryoablation acts directly on the tumor mass and may address local mechanical, inflammatory, and perineural components of pain at their source. This rationale supported the selection of cryoablation in patients in whom local tumor burden was considered a relevant contributor to symptoms.

The mechanism of pain relief after tumor cryoablation is likely multifactorial. Local treatment may reduce mass effect, mechanical irritation, peritumoral inflammation, and pain-related mediator release. In addition, freezing may exert a local cryoanalgesic effect on tumor-infiltrated neural structures [6,16,17]. In PDAC, where perineural invasion is an important contributor to pain, these mechanisms may contribute to symptom relief after treatment [6].

One relevant aspect of this experience is that the procedure was conceived as a tumor-directed palliative intervention rather than as an indirect neural pain procedure. However, imaging findings should be interpreted cautiously. The observed reduction in the contrast-enhancing tumor component was assessed descriptively and was not based on standardized response criteria. Therefore, these findings should be considered exploratory evidence of a local cytoreductive effect, reflected by reduction in the contrast-enhancing component, rather than proof of formal tumor response or oncologic local control.

One of the most relevant findings of this study is the reduction in analgesic requirements. All patients were receiving opioid-based therapy at baseline. During follow-up, 45.5% achieved complete opioid discontinuation, while the remaining 54.5% showed a reduction in opioid requirement without the need for escalation. This aspect is clinically meaningful, since chronic opioid treatment is frequently associated with relevant side effects, including nausea, constipation, sedation, and reduced overall quality of life [6,8]. In this setting, the ability to obtain pain relief together with a lower analgesic requirement may represent an important practical advantage of tumor-directed cryoablation. This finding is clinically relevant, although it should be interpreted descriptively given the absence of a control group and the qualitative assessment of analgesic requirements. Nevertheless, this qualitative categorization provides a pragmatic real-world perspective on analgesic needs in a palliative setting.

Supportive clinical outcomes in our series further support the palliative value of this approach. Weight recovery was documented in two-thirds of patients with baseline weight loss, half of the patients with sleep disturbance reported improvement in sleep quality, and ECOG performance status improved by at least one level in more than one-third of the cohort at 1 month. Although these endpoints were not assessed using validated quality-of-life instruments, they suggest that pain reduction after cryoablation may translate into broader clinical benefit. This is particularly relevant in PDAC, where progressive pain, cachexia, fatigue, and functional decline often coexist and strongly affect patient management [4,6].

The safety profile observed in our experience was favorable. No major complications occurred, and minor adverse events were limited and self-limited, including transient abdominal pain, nausea, and one conservatively managed pleural effusion. No clinically overt post-procedural pancreatitis, bleeding, duodenal injury, or infection was recorded. In the context of the available literature, these findings may compare favorably with the complication rates reported for other pancreatic ablative techniques, particularly irreversible electroporation and radiofrequency ablation [12,13,14,15,31]. In our opinion, this favorable safety profile is partly related to careful patient selection and to a deliberately conservative technical strategy, aimed at intratumoral cytoreduction rather than wide-margin ablation. In addition, patients with obvious duodenal invasion were excluded, and in two cases a transgastric route was adopted without complications when this represented the safest access trajectory.

Imaging findings should be interpreted in light of the palliative intent of the procedure. At 1-month contrast-enhanced CT, residual enhancement was still present in most cases, although with a marked reduction in enhancing tumor burden; complete absence of residual enhancement was observed only in a minority of patients. This was expected, since the objective of treatment was not radical ablation but symptom-oriented local treatment while preserving safety. Interestingly, meaningful pain relief was observed in all patients regardless of the presence of residual enhancement, suggesting that complete radiologic ablation may not be necessary to obtain clinically relevant palliation. In this sense, our results support the concept that even partial local cytoreduction may be sufficient to reduce tumor-related pain in selected patients [16,17].

Survival was not a primary endpoint of the present study, and survival outcomes were reported descriptively only. The small sample size, retrospective design, patient selection, and frequent use of previous or concomitant systemic treatments do not allow any conclusion regarding survival benefit or oncologic efficacy attributable to cryoablation. Observed survival proportions at 6 and 12 months were 90.9% and 72.7%, respectively, but these data should be interpreted cautiously and cannot be isolated from the overall multidisciplinary management of the disease [2,3,4]. Therefore, the main value of cryoablation in this setting should be considered palliative rather than survival-oriented.

This study has several limitations. First, it is a retrospective single-center pilot series with a very small sample size, which limits statistical power and generalizability. Second, there was no control group, and therefore the observed clinical benefits cannot be causally attributed to cryoablation or directly compared with those achievable by standard medical management, celiac plexus neurolysis, radiotherapy, systemic therapy, or other palliative approaches. In addition, the observed reduction in pain and analgesic requirement may have been influenced by several confounding factors, including the natural variability of cancer-related pain, concomitant oncologic and supportive treatments, patient selection, and possible placebo- or procedure-related effects. Moreover, the cohort was clinically heterogeneous, with a predominance of metastatic disease and variability in previous and concomitant oncologic treatments. This heterogeneity may have influenced baseline symptom burden, pain evolution, functional status, and clinical outcomes, and the small sample size precluded formal subgroup analyses. Potential selection bias should also be acknowledged, as patients were selected for cryoablation based on clinical condition, symptom burden, technical feasibility, anatomical suitability, and multidisciplinary judgment. Third, although pain response was clearly documented, analgesic requirement was categorized descriptively rather than quantified using standardized morphine-equivalent dosing. Fourth, supportive clinical outcomes, including sleep quality, body weight changes, and ECOG performance status, were retrospectively derived from clinical records rather than from dedicated validated quality-of-life instruments. Therefore, these outcomes should be interpreted as descriptive and supportive rather than definitive measures of broader clinical benefit. Fifth, imaging outcomes were assessed descriptively and not according to standardized radiologic response criteria such as RECIST, mRECIST, or Choi criteria. Therefore, changes in residual enhancement or lesion morphology should be interpreted as exploratory imaging findings rather than formal evidence of tumor response or oncologic local control. Finally, patient attrition due to progression and death reduced the number of evaluable subjects at later follow-up time points, introducing a potential attrition bias, as the most favorable 12-month outcomes were necessarily derived from surviving and clinically evaluable patients.

Despite these limitations, the present study provides preliminary evidence that percutaneous CT-guided cryoablation may have a relevant palliative role in selected patients with painful unresectable PDAC. The combination of rapid pain relief, reduction in opioid requirement, acceptable short-term safety, and supportive clinical improvement suggests that this technique may be considered as a complementary option within a multidisciplinary palliative strategy, particularly in patients with persistent tumor-related pain despite medical treatment or in cases where other interventional pain procedures are expected to provide limited benefit [9,26].

From a future-perspective standpoint, recent reviews on thermal ablation in pancreatic cancer and emerging technological developments further support the need to investigate cryoablation within structured clinical protocols [12,23]. Prototype cryoablation systems designed for potential compatibility with endoscopic ultrasound guidance may improve access to pancreatic lesions and procedural monitoring in the future, although current evidence remains preliminary and largely preclinical [24]. In addition, the potential immunomodulatory effects of cryoablation, including tumor antigen release and stimulation of antitumor immune responses, provide a biological rationale for future studies evaluating combinations with systemic therapies or immunotherapy [25]. However, these translational perspectives remain exploratory and should not be inferred from the present clinical series, which was designed to assess clinical palliation rather than immunologic or technological endpoints.

Further prospective studies with larger cohorts are needed to better define the role of pancreatic cryoablation in this setting. Future investigations should include standardized pain instruments, quantitative assessment of opioid consumption using morphine-equivalent daily dose, validated quality-of-life questionnaires, structured imaging follow-up, and comparison with other palliative interventions such as celiac plexus neurolysis, celiac plexus radiosurgery, and cryoneurolysis of the splanchnic nerves [9,10,11,26,27,28,29]. In addition, future studies should explore whether quantitative imaging biomarkers, radiomics-based features, or artificial intelligence-supported imaging analysis may help identify patients most likely to benefit from tumor-directed cryoablation and better characterize local treatment effect over time. The possible integration of cryoablation with other local or systemic strategies also deserves further study [15,17,25,32].

## 5. Conclusions

In conclusion, percutaneous CT-guided cryoablation appears to be a feasible and promising palliative option for pain management in selected patients with unresectable PDAC. It may deserve further prospective evaluation and could be considered, in carefully selected cases, within a multidisciplinary palliative treatment strategy.

## Figures and Tables

**Table 1:** Baseline patient, disease, and tumor characteristics before percutaneous CT-guided cryoablation.

Variable	Value
Number of patients	11
Sex	10 women, 1 man
Age, years	72.36 ± 9.19
Histology	11/11 PDAC (100%)
Primary pancreatic tumor	11/11 (100%)
Disease extent at cryoablation	
Locally advanced unresectable	2/11 (18.2%)
Metastatic unresectable	9/11 (81.8%)
Prior surgery	1/11 (9.1%)
Prior/Ongoing chemotherapy	8/11 (72.7%)
Prior radiotherapy	2/11 (18.2%)
Tumor location	
Pancreatic head	8/11 (72.7%)
Pancreatic body/tail region	3/11 (27.3%)
Maximum axial tumor diameter, mm	39.36 ± 9.81
Vascular infiltration	8/11 (72.7%)
Baseline ECOG performance status	
ECOG 2	5/11 (45.5%)
ECOG 3	6/11 (54.5%)
Baseline weight loss	9/11 (81.8%)
Baseline sleep disturbance	6/11 (54.5%)
Baseline VAS pain score	6.72 ± 1.56
Baseline opioid-based analgesic therapy	11/11 (100%)

Data are presented as n (%) or mean ± standard deviation, unless otherwise specified. PDAC: pancreatic ductal adenocarcinoma; ECOG: Eastern Cooperative Oncology Group; VAS: visual analogue scale.

**Table 2:** Procedural, clinical, and imaging outcomes after percutaneous CT-guided cryoablation.

Variable	Value
Procedural outcomes	
Technical success	11/11 (100%)
Patients with minor adverse events	3/11 (27.3%)
Total minor adverse events	5
Transient abdominal pain	2
Nausea	2
Pleural effusion	1
Major complications	0
Pain and analgesic outcomes	
Baseline VAS score	6.72 ± 1.56
VAS at 1 month	3.45 ± 1.44 (*n* = 11)
VAS at 3 months	2.54 ± 1.29 (*n* = 11)
VAS at 6 months	2.27 ± 1.43 (*n* = 10)
VAS at 12 months	1.60 ± 1.07 (*n* = 8)
≥3-point VAS reduction at 1 month	11/11 (100%)
Complete opioid discontinuation	5/11 (45.5%)
Opioid reduction without discontinuation	6/11 (54.5%)
Supportive clinical outcomes	
Weight recovery > 5%	6/9 (66.7%)
Sleep improvement	3/6 (50.0%)
ECOG improvement at 1 month	4/11 (36.4%)
ECOG stable at 1 month	6/11 (54.5%)
ECOG worsened at 1 month	1/11 (9.1%)
Imaging and survival outcomes	
Residual enhancement at 1 month	9/11 (81.8%)
No residual enhancement at 1 month	2/11 (18.2%)
Estimated reduction in enhancing tumor burden	50–80%

## Data Availability

The data presented in this study are available on reasonable request from the corresponding author. The data are not publicly available due to privacy and ethical restrictions.

## Abbreviations

The following abbreviations are used in this manuscript:

CT	computed tomography
DWI	diffusion-weighted imaging
ECOG	Eastern Cooperative Oncology Group
INR	international normalized ratio
MRI	magnetic resonance imaging
PDAC	pancreatic ductal adenocarcinoma
VAS	visual analogue scale
